# Efficacy, precision and ease of 3D-printed versus conventional endodontic instruments compared to conventional stainless steel and nickel-titanium files in root canal preparation

**DOI:** 10.6026/973206300213031

**Published:** 2025-09-30

**Authors:** Ishika Chatterjee, Supriya Patil, Prachi Joshi, Debkant Jena, Maneesha Das, Amrutha Sathianath

**Affiliations:** 1 Department of Conservative Dentistry & Endodontics, Kalinga Institute of Dental sciences, KIIT University, Patia, Bhubaneswar, Odisha, India; 2 Department of conservative and Endodontics, H.K.E's, S Nijalingappa Institute of Dental sciences and Research, Kalaburgi, Karnataka, India; 3 Department of Conservative Dentistry and Endodontics, Ajeenkya D.Y.Patil Dental School, Pune, Maharashtra, India; 4 Department of Conservative Dentistry and Endodontics, Institute of Dental Sciences, Siksha 'O' Anusandhan deemed to be University, Kalinga Nagar, Bhubaneswar-751003, Odisha, India; 5 Department of Conservation Dentistry and endodontist, KMCT Dental College Campus, Mukkam, Manassery, Kerala, India

**Keywords:** 3D-printed endodontic files, root canal preparation, NiTi files, stainless steel files, endodontic instrumentation

## Abstract

The performance of 3D-printed endodontic instruments against traditional stainless steel and NiTi files in root canal preparation. Hence, a total of 135 patients requiring single-rooted canal treatment were enrolled and randomized into three groups. Parameters assessed included canal shaping efficacy, procedural accuracy and operator handling comfort. 3D-printed instruments demonstrated comparable shaping efficiency and significantly improved precision and ease of use. These findings suggest promising potential for 3D-printed tools in clinical endodontics.

## Background:

Root canal preparation is a critical step in endodontic therapy, directly influencing treatment success by ensuring thorough debridement and optimal canal shaping [1]. Traditional instruments, primarily stainless steel and nickel-titanium (NiTi) files, have been the cornerstone of root canal instrumentation for decades [2]. Stainless steel files offer rigidity and control but are limited in curved canals due to their lack of flexibility, increasing the risk of canal transportation or ledging [3]. NiTi files, with their superior flexibility and shape memory, provide better conformation to canal curvatures and reduced procedural errors; however, they are associated with challenges such as instrument separation, higher cost and limited customization [4]. With the advent of advanced digital technologies, three-dimensional (3D) printing has emerged as a promising tool in medical and dental applications. In endodontics, 3D printing enables the creation of highly customized instruments with precise geometries, tailored tapering and potentially optimized cutting efficiency [5]. These advantages may translate into improved clinical outcomes, enhanced operator comfort and reduced procedural complications [6]. However, empirical data on the performance of 3D-printed endodontic files remain sparse [7]. Therefore, it is of interest to assess and compare the efficacy, precision and ease of use of 3D-printed endodontic instruments with that of conventional stainless steel and NiTi files in root canal preparation, focusing on clinical outcomes and user experience.

## Materials and Methods:

This prospective randomized controlled study was conducted on 135 patients aged 18-60 years, each presenting with a single-rooted tooth requiring root canal treatment. Patients were randomly allocated into three equal groups (n=45 per group): Group A treated with 3D-printed endodontic files, Group B with conventional stainless steel files and Group C with standard nickel-titanium (NiTi) rotary files. All procedures were performed by experienced endodontists under rubber dam isolation. Working length was established using an electronic apex locator and confirmed radiographically. Canal preparation in Group A utilized custom-designed 3D-printed files fabricated via selective laser melting with a proprietary nickel-titanium alloy blend. Group B employed hand stainless steel K-files using a step-back technique and Group C used rotary NiTi files following manufacturer's instructions. Primary outcomes included shaping efficacy (measured by canal taper and centering ability on pre- and post-instrumentation CBCT scans), procedural precision (evaluated by incidence of canal aberrations such as ledges, transportation, or perforations) and ease of use (assessed via operator questionnaire using a 5-point Likert scale for handling comfort, cutting efficiency and fatigue). Time taken for canal preparation was also recorded. Data were analyzed using appropriate statistical tests with significance set at p<0.05. Ethical approval was obtained and all participants provided informed consent.

## Results:

The results below summarize the comparative performance of 3D-printed endodontic instruments, stainless steel files and nickel-titanium (NiTi) files in root canal preparation. Key outcomes include shaping efficacy, procedural precision, ease of use and preparation time across the three groups. Table 1 (see PDF) shows age and gender distribution were similar across the three groups, indicating well-matched cohorts for comparison. Table 2 (see PDF) shows the 3D-printed instrument group showed a significantly reduced preparation time compared to stainless steel files and was comparable to NiTi files. Table 3 (see PDF) shows 3D-printed files achieved significantly higher taper conformity compared to stainless steel files and similar to NiTi files. Table 4 (see PDF) shows 3D-printed instruments demonstrated superior centering ability compared to stainless steel and were comparable to NiTi files. Table 5 (see PDF) shows the 3D-printed group had fewer procedural errors than stainless steel files, similar to the NiTi group. Table 6 (see PDF) shows operators rated 3D-printed files higher in cutting efficiency than stainless steel files and comparable to NiTi files. Table 7 (see PDF) shows the 3D-printed files scored significantly better for handling comfort than stainless steel and similarly to NiTi files. Table 8 (see PDF) shows the Use of 3D-printed files resulted in less operator fatigue than stainless steel and was comparable to NiTi files. Table 9 (see PDF) shows the 3D-printed instruments exhibited low deformation/failure rates, similar to NiTi files and better than stainless steel files. Table 10 (see PDF) shows no significant difference was observed among groups regarding postoperative pain.

## Discussion:

The present study demonstrates that 3D-printed endodontic instruments offer comparable efficacy and precision to conventional nickel-titanium (NiTi) files, while outperforming stainless steel files in several critical parameters. The significantly reduced preparation time observed with 3D-printed instruments aligns with their enhanced cutting efficiency and better canal centering ability, suggesting improved design adaptability that allows for more effective shaping of root canals [8]. These advantages are particularly relevant considering that stainless steel files exhibited longer preparation times and higher incidences of procedural errors, such as ledging and canal transportation [9]. Operator feedback further supports the superiority of 3D-printed instruments in terms of handling comfort and reduced fatigue, factors that may contribute to better clinical outcomes and enhanced user experience [10]. The low deformation and failure rates recorded with 3D-printed files indicate that despite being a novel technology, they possess adequate durability comparable to NiTi files, which have long been favored for their flexibility and resistance to fracture [11]. Although postoperative pain scores did not differ significantly among groups, the overall procedural success rates were notably higher for the 3D-printed and NiTi groups, reflecting their precision and safer canal preparation. Operator preference data reinforce the clinical acceptability of 3D-printed instruments, with a majority favoring them over traditional options [12]. These findings highlight the potential for 3D printing technology to revolutionize endodontic practice by offering customizable, efficient and user-friendly tools. However, further long-term clinical studies are warranted to evaluate the performance of 3D-printed instruments across diverse canal anatomies and to assess their cost-effectiveness and integration into routine practice.

## Conclusion:

3D-printed endodontic instruments demonstrate comparable efficacy and precision to nickel-titanium files while offering improved ease of use and reduced preparation time compared to stainless steel files. Thus, we show promising durability and operator satisfaction, making them a viable alternative in root canal preparation. Further clinical studies are needed to confirm long-term benefits and cost-effectiveness.
